# OsCERK1 Contributes to Cupric Oxide Nanoparticles Induced Phytotoxicity and Basal Resistance against Blast by Regulating the Anti-Oxidant System in Rice

**DOI:** 10.3390/jof9010036

**Published:** 2022-12-26

**Authors:** Ya Chen, Zhiquan Liu, Shuai Meng, Zhenan Shen, Huanbin Shi, Jiehua Qiu, Fucheng Lin, Shu Zhang, Yanjun Kou

**Affiliations:** 1 State Key Laboratory of Rice Biology, China National Rice Research Institute, Hangzhou 311400, China; 2 College of Agriculture and Biotechnology, Zhejiang University, Hangzhou 310058, China; 3 State Key Laboratory for Managing Biotic and Chemical Threats to the Quality and Safety of Agro-Products, Institute of Plant Protection and Microbiology, Zhejiang Academy of Agricultural Sciences, Hangzhou 310021, China; 4 Key Laboratory of Integrated Pest Management on Crops in Central China, Ministry of Agriculture, Wuhan 430064, China; 5 Institute for Plant Protection & Soil Fertilizer, Hubei Academy of Agricultural Sciences, Wuhan 430064, China; 6 Hubei Key Laboratory of Crop Disease, Insect Pests and Weeds Control, Wuhan 430064, China

**Keywords:** *Oryza sativa*, *Magnaporthe oryzae*, rice blast, basal resistance, cupric oxide nanoparticles, ROS

## Abstract

CuO NPs (cupric oxide nanoparticles) are widely used in various fields due to their high electrical conductivity, electronic correlation effect, and special physical property. Notably, CuO NPs have good application prospects in agricultural production because of its antifungal activity to prevent crop diseases. However, the increasing release of CuO NPs into the environment has resulted in a serious threat to the ecosystem, including plants. Previous studies have reported the toxicity of CuO NPs on rice, but little is known about the underlying molecular mechanisms or specific genes involved in the response to CuO NPs. In this study, we found that the rice well-known receptor Chitin Elicitor Receptor Kinase 1 (OsCERK1), which is essential for basal resistance against pathogens, is involved in CuO NPs stress in rice. Knockout of *OsCERK1* gene resulted in enhanced tolerance to CuO NPs stress. Furthermore, it was revealed that OsCERK1 reduces the tolerance to CuO NPs stress by regulating the anti-oxidant system and increasing the accumulation of H_2_O_2_ in rice. In addition, CuO NPs treatment significantly enhances the basal resistance against *M. oryzae* which is mediated by OsCERK1. In conclusion, this study demonstrated a dual role of OsCERK1 in response to CuO NPs stress and *M. oryzae* infection by modulating ROS accumulation, which expands our understanding about the crosstalk between abiotic and biotic stresses.

## 1. Introduction

With the application of nanotechnology in many industries, it increases the potential exposure level of nanoparticles in the environment, and also raises concerns about their accumulation in crops and humans through crop consumption. CuO NPs (cupric oxide nanoparticles) possess high electrical conductivity, electronic correlation effect, and special physical and chemical properties, so they are widely used in gas sensors, catalysts, high temperature conductors, solar energy converters, cosmetics, medicine, and other fields [1]. With the wide use of CuO NPs, the exposure of CuO NPs to the environment and humans has increased rapidly [2]. Studies have shown that CuO NPs were detected in water, soil, and air [3]. After absorption, CuO NPs cause various degrees of toxicity to plants and animals [4,5,6], and even induce liver and intestine cancer in humans [3], which seriously threatens human health. 

In plants, exposure to high concentration of CuO NPs reduces plant growth, germination rates, root elongation, and biomass accumulation. For instance, CuO NPs inhibit the root and shoot growth in Indian mustard [7]. Moreover, CuO NPs at the concentration of 500 mg/kg inhibit the growth of maize [8]. Different from the concentration in maize, the concentration of CuO NPs exceeding 10 mg/L could strongly inhibit the biomass accumulation in cotton [9]. In addition, CuO NPs reduce the frond number, frond surface area, and dry weights of whole plants in duckweed [10]. Further studies showed that the phytotoxicity caused by CuO NPs on plants is related to oxidative stress, DNA damage, and photosynthesis interferences [11]. Rice (*Oryza sativa*) is a major crop plant and a staple food feeding about half of the world’s population. It contributes the bulk of calories, protein, and most of trace elements for people [12]. Recent studies demonstrated that the accumulation of CuO NPs strongly inhibits the growth and reduces seed germination rate in rice [13]. However, the molecular mechanism of rice in response to CuO NPs stress is still unclear.

Recent studies showed that CuO NPs have good application prospects in agricultural production with certain antifungal activity. CuO NPs inhibit the growth of *Fusarium oxysporum*, which causes Fusarium wilt of watermelon, thereby increasing the yield of watermelon [14]. In addition, CuO NPs protects cucumber from cucumber downy mildew [15]. Similarly, CuO NPs possess greater antifungal activity towards *Aspergillus niger* and *Mucor piriformis* [16]. *Magnaporthe oryzae*, which is the causal agent of rice blast disease, decreases rice production by an amount enough to feed 60 million people every year [17]. It infects all the developmental stages of rice plants and causes symptoms on the leaf, collar, neck, and panicle. In response to *M. oryzae* infection, basal resistance is activated with the expression of *pathogenesis-related (PR)* genes, such as *OsPR1a*, *OsPR10*, *OsPAL,* and *OsNAC4*, and ROS (reactive oxygen species) accumulation are induced in rice [18,19]. Currently, it is unclear whether CuO NPs protect rice from blast fungus infection.

CERK1 (Chitin Elicitor Receptor Kinase 1) is a transmembrane receptor with lysine motifs (LysMs), which triggers basal resistance in plants by perceiving chitin from pathogenic fungi [20]. In *Arabidopsis thaliana*, AtCERK1 binds chitin to form a homologous dimer, and then its kinase domain is phosphorylated to transmit the chitin signals from extracellular to intracellular [21,22]. In rice, OsCERK1 plays a central role in chitin-triggered basal resistance against *Magnaporthe oryzae*. Upon perceiving *M. oryzae*, OsCERK1 interacts with the LysM receptor-like kinase OsCEBiP, which directly binds to chitin, to form a heteropolymeric complex [22]. Subsequently, OsCERK1 activates downstream signaling components and induces ROS burst, MAPK (mitogen-activated protein kinase) activation, and expression of PR genes to enhance basal resistance in rice [23]. Recent studies revealed that OsCERK1 is also required for the perception of short-chain chitin oligomers during initiation of arbuscular mycorrhizal symbiosis [24]. In addition to participating in the resistance against fungal pathogens and establishing symbiosis, CERK1 interacts with ANN1 (ANNEXIN 1), which is a NaCl-induced calcium-permeable channel, to positively regulate abiotic salinity stress tolerance in *Arabidopsis* [25]. At present, it is unclear whether OsCERK1 participates in the response to other abiotic stresses.

In our previous study on environmentally regulated blast resistance, we noticed that the expression level of basal resistance gene *OsCERK1* was induced by environmental changes/abiotic stresses [13]. Recent studies have shown that CuO NPs emerge to be a novel environmental stress and pose a threat to the ecosystem by causing toxicity to various plants with more CuO NPs released into the environment [26,27]. With the aim of elucidating the molecular mechanism of rice responding to abiotic CuO NPs stress, the role of OsCERK1 in the phytotoxicity of CuO NPs was investigated in this study. It was shown that OsCERK1 is involved in the responses to CuO NPs stress in rice. Furthermore, we focused on the biological function of OsCERK1 responding to CuO NPs stress and explored whether there is crosstalk between the response to CuO NPs and disease resistance in rice. The results demonstrated that OsCERK1 functions as a core module in biotic and abiotic stresses through modulating ROS accumulation.

## 2. Materials and Methods

### 2.1. Characterization of CuO NPs

**Figure 1:**
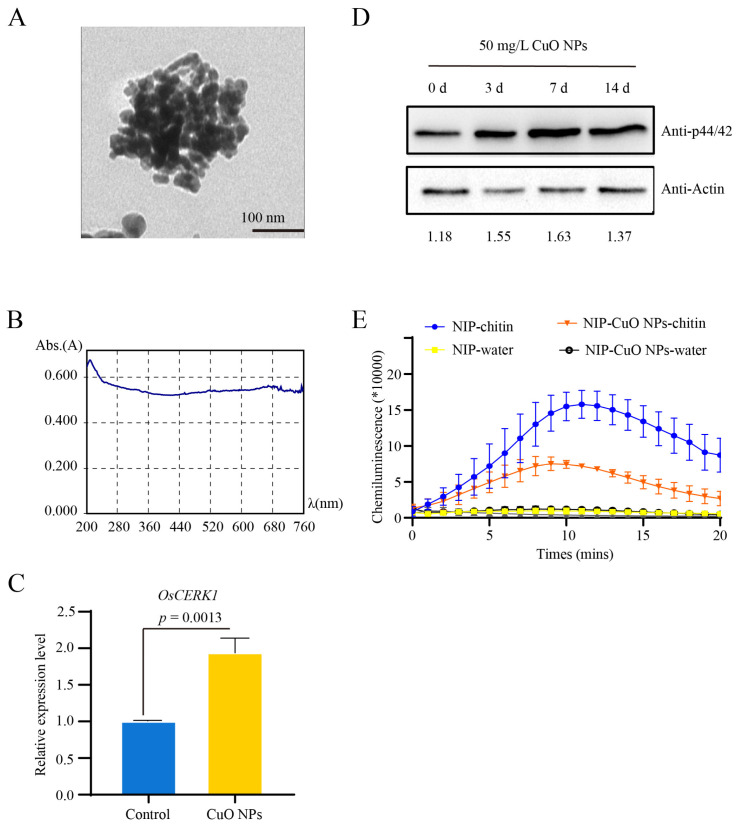
The well-known receptor kinase gene *OsCERK1* responds to CuO NPs stress in rice. (**A**) Characterization of the CuO NPs used in this study. (**B**) The absorption spectrum of the CuO NPs suspension. The absorption peak was about 210 nm. (**C**) Transcript abundance of *OsCERK1* in the rice seedlings treated with CuO NPs. (**D**) The MAPKs activities in the rice seedlings treated with CuO NPs were higher than those of the control. The total proteins were extracted from Nipponbare (NIP) seedlings treated with CuO NPs at 3, 7, and 14 days to perform western blot assay with anti-phospho-p44/42 anti-body. The relative intensities of the blots were quantified by ImageJ with actin as an internal control. (**E**) Reactive oxygen species (ROS) generation was effectively induced by chitin in the CuO NPs treated rice seedlings. The bars are shown as means ± SD of three replications. The student’s *t*-test was used to analyze the data and generate *p* value.

CuO NPs (copper II oxide nanoparticles, purity 99.9%) were purchased from Shanghai Chaowei Nano Technology Co., Ltd. The morphology and particle size distribution of the CuO NPs were evaluated by transmission electron microscopy (TEM; JEOL Ltd., JEM-1010, Tokyo, Japan) and biospectrometer (Eppendorf, Hamburg, Germany) after sonication of the nanoparticle suspension using a transonic 420 (Kedao, Shanghai, China) for 30 min. The images of TEM (Figure 1A) indicated that CuO NPs were in spherical shape with an average diameter of 40 nm. The absorption peak of the CuO NPs suspension was at 210 nm.

### 2.2. Culture and CuO NPs Treatment of Rice Seedlings

The wild-type *Oryza sativa* subsp. *Japonica* cultivars Nipponbare and TP309, and transgenic rice plants were used as experimental materials. The *oscerk1* mutant, which was generated in Nipponbare background using CRISPR/cas9 system (clustered regularly interspaced short palindromic repeats/CRISPR-associated protein 9) as previously described [28].

Rice seeds were immersed in the 9 cm dishes with water at 37 °C for 1 day in the dark, then germinated on the wet paper for 4–5 days at 25 °C under 16 h light/8 h dark photoperiod to obtain uniform rice seedlings. For hydroponics, the seedlings were planted into plastic pots (sph-96, Shuoke, Hangzhou, China) with Yoshida nutrient solution (pH 5.5–5.8) (NSP1040, Coolaber, Beijing, China).

For CuO NPs treatment, the rice seedlings were cultured in nutrient solutions with 0 mg/L and 50 mg/L CuO NPs, which was known to significantly inhibit the growth of rice [29]. After 14 days, the roots and shoots of rice seedlings were collected to quantitatively determine the plant height, fresh biomass, and indexes of the anti-oxidative system. All of these experiments were performed in triplicate.

### 2.3. Rice Seedling Infection Assay

Rice seedling infection assays were performed with 5 × 10^5^ mL^−1^ conidial suspension as previously described [30]. The *M. oryzae* strain En2-2, which was isolated from a paddy field in Enshi, Hubei province, China [31], was cultured on OA plates at 28 °C in dark conditions for 7 days to collect conidia [32]. After spray inoculation with conidia, the rice seedlings were cultured under dark condition for 24 h, and then transferred into an incubator with a 12 h light/12 h dark cycle for 6 days at 22 °C. Disease symptoms of infection assays were assessed on 7 days post inoculation and shown by the images. The relative lesion area was calculated by ImageJ. The infection assays were repeated three times with similar results

### 2.4. Quantitative RT-PCR Analysis

Total RNA was extracted from the rice leaves using Trizol (Invitrogen, 15596-026) and reversely transcribed using M-MLV enzyme (Beyotime, D7176L-1) according to the manufacturer’s protocol. Quantitative real-time PCR (qRT-PCR) was applied to determine relative gene expression levels using Hieff^®^ qPCR SYBR Green Master Mix (Yeasen Biotech, Shanghai, China). The primers for qRT-PCR analyses were shown in the Supplemental Table S1. The *OsUbiquitin* gene (LOC_Os03g13170) was used as an internal control for normalization. The relative transcript abundances were calculated with the 2^−∆∆CT^ method based on the abundance levels in control samples.

### 2.5. MAPK Assay

The rice seedlings were collected after CuO NPs treatment for 1, 3, 7, and 14 days, then ground in liquid nitrogen and homogenized in an extraction buffer containing 25 mM Tris-HCl (pH 7.5), 150 mM NaCl, 5% glycerol, 1% NonidetP-40, 1 mM EDTA, 1 mM PMSF, and 1× protease inhibitor cocktail (Roche, Basel, Switzerland). Then, the total proteins were resolved in 10% SDS-polyacrylamide gel. Phosphorylation of MAPK proteins was detected by immunoblotting with anti-phospho-p44/42 MAPK antibody (Cell Signaling Technology, 4370s). The Actin protein was used as a control. Similar results were obtained from two biological replicates.

### 2.6. Measurement of ROS

For measurement of ROS, the upper leaves of the treated rice seedlings were cut to 0.25 cm^2^ circles, and then floated in sterilized water overnight at room temperature to recover from wounding stress. The leave circles were placed in a 1.5 mL tube containing 1 μL horseradish peroxidase, 100 μL L-012 (Wako, Tokyo, Japan), and elicitor (8 nM hexa-N566 acetyl-chitohexaose). Double distilled H_2_O served as control. The luminescence was recorded continuously at 10 s intervals for 20 min by a Glomax 20/20 luminometer (Promega, Beijing, China). Three replications were carried out with similar results.

### 2.7. Measurement of Hydrogen Peroxide (H_2_O_2_) Content

The levels of H_2_O_2_ were examined according to the commercial kits (Solarbio, Beijing, China) with the manufacturer’s protocol [33]. About 0.1 g of rice seedlings was ground and blended in 500 μL of lysate. Then the homogenate was centrifuged at 4 °C for 10 min at 8000× *g*. The supernatant was mixed with the same account of H_2_O_2_ detection reagent to detect the absorbance at 415 nm. The standard curve was utilized to analyze the content of H_2_O_2_. And H_2_O_2_ content was calculated according to the manufacturer’s instructions. Three replications were carried out with similar results.

### 2.8. Measurement of Activities of Anti-Oxidant Enzymes

The anti-oxidant enzyme activities, including peroxidase (POD), superoxide dismutase (SOD), and catalase (CAT), were quantified using the commercial kits (Solarbio, Beijing, China) as previously described [33]. Approximately 0.1 g nitrogen ground sample was added into 1 mL precooled corresponding extraction solution in the kit. And the absorbance of the reaction products was detected. The activities of anti-oxidant enzymes were standardized as following descriptions: one unit of activity of SOD was defined as the amount of enzyme that caused the inhibition of photoreduction of 4-nitroblue tetrazolium chloride by 50%. One unit of POD activity was defined as the amount of enzyme that caused an increase in absorbance at 470 nm of 0.001 per minute. One unit of CAT activity was defined as the amount of enzyme that caused an increase in absorbance at 470 nm of 0.001 per minute. Similar results were obtained from three replications.

### 2.9. Investigation of Physiological Features

The rice seedlings treated with 50 mg/L CuO NPs for 14 days were collected. After thoroughly rinsing with sterilized water, the physiological features of the rice seedlings, including shoot length, root length, and root number were determined. The fresh biomass was measured after drying the surface of rice with filter paper. The data was shown as the mean with standard deviation (SD) based on ten replications. The relative inhibition rate of fresh biomass was calculated based on the formula: inhibition rate (%) = [fresh biomass (control) − fresh biomass (CuO NPs)]/fresh biomass (control) × 100%. Similarly, the relative inhibition rates of shoot length, root length, root number, and the third leaf length were calculated according to the formula.

### 2.10. Data Analysis

Relative expression values were calculated using the 2^−∆∆*C*T^ method. The Students’ *t*-test was used for performing all statistical analyses and generating *p* value. The data was shown as mean with SD based on at least three repetitions.

## 3. Results

### 3.1. Basal Resistance Gene OsCERK1 Is Involved in Response to CuO NPs Stress in Rice

To investigate whether *OsCERK1* is involved in the response to CuO NPs stress, the expression level of *OsCERK1* in the rice seedlings treated with 50 mg/L CuO NPs was detected. The results showed the expression level of *OsCERK1* in the CuO NPs-treated rice seedlings was significantly higher than that in the control (Figure 1B). It was known that OsCERK1 exerts an important role in mediating chitin-induced ROS burst and MAPK activation [18]. To further investigate whether the activity of OsCERK1 was changed in response to CuO NPs in rice, the MAP kinases activity, and the ROS accumulation in the CuO NPs treated rice seedlings were determined. The results showed that the phosphorylation levels of MAP kinases were higher in the seedlings with CuO NPs treatment than that without CuO NPs (Figure 1C). Meanwhile, chitin-induced ROS accumulation was increased in the seedlings under CuO NPs stress condition (Figure 1D). The increased MAP kinases activity and ROS accumulation may be caused by the up-regulated expression of *OsCERK1* upon CuO NPs treatment. All these results suggested that the LysM receptor-like kinase OsCERK1 is involved in the response to CuO NPs stress in rice.

### 3.2. OsCERK1 Regulates Phytotoxicity of CuO NPs Stress to Rice

**Figure 2:**
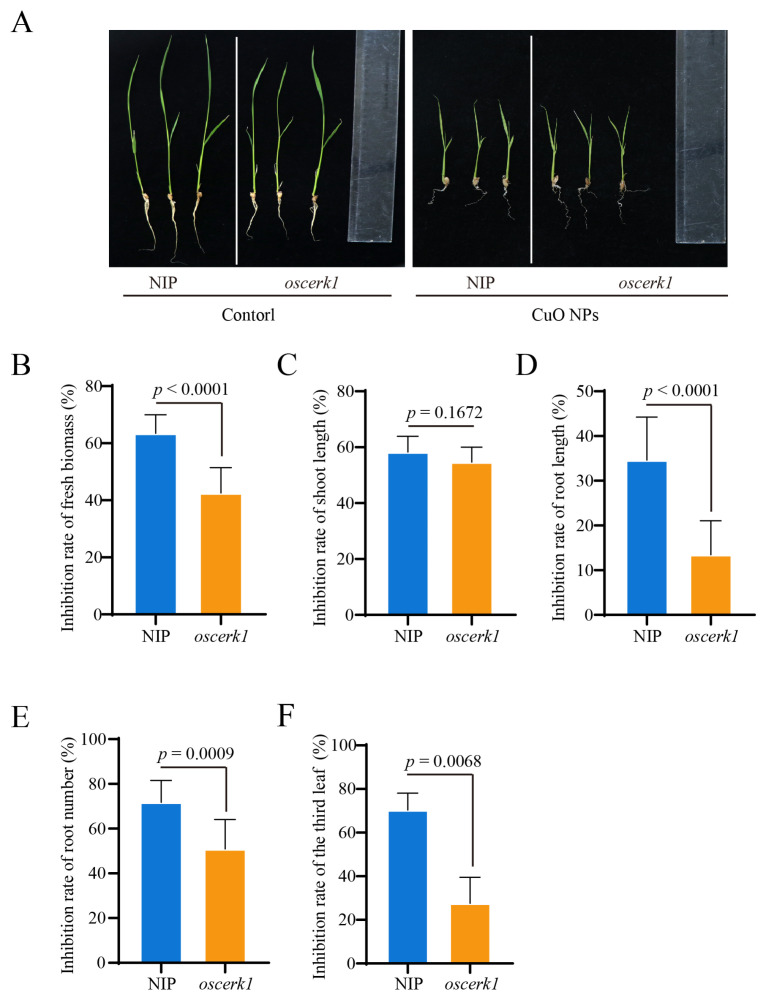
OsCERK1 regulates the tolerance of rice to CuO NPs stress. (**A**) The growth of the *oscerk1* mutant and its wild-type Nipponbare (NIP) treated with CuO NPs for 14 days. The relative inhibition rates of fresh biomass (**B**), shoot length (**C**), root length (**D**), root numbers (**E**), and the third leaves’ length (**F**) of the *oscerk1* mutants and its wild-type treated with CuO NPs for 14 days. The relative inhibition rate of fresh biomass was calculated based on the formula: inhibition rate (%) = [fresh biomass (control) − fresh biomass (CuO NPs)] / fresh biomass (control) × 100%. Similarly, the relative inhibition rates of shoot length, root length, root number, and the third leaf length were calculated according to the formula. The bars are shown as means ± SD of ten replications. The student’s *t*-test was used to analyze the data and generate *a p* value.

To investigate the biological role of *OsCERK1* in the response to CuO NPs stress in rice, we determined the effect of CuO NPs on the *oscerk1* mutant, which was created using CRISPR-Cas9 system, and its wild-type [28]. The growth of rice seedlings was significantly inhibited by CuO NPs (Figure 2A). Upon CuO NPs treatment for 14 days, the fresh biomass of the wild-type was significantly reduced by 64%, while that in the *oscerk1* mutant only decreased by 43% (Figure 2B). Moreover, the growth of the third leaf (the white arrow) of the wild-type was inhibited by CuO NPs stress, while the inhibitory phenotype was partially restored by the knockout of *OsCERK1* gene (Figure 2A,F). Furthermore, CuO NPs treatment strongly inhibited root growth in rice (Figure 2D,E). After CuO NPs treatment, the root lengths of the *oscerk1* mutants were significantly longer than those of the wild-type (Figure 2D), and the root numbers of the *oscerk1* mutants were more than those of the wild-type (Figure 2E), suggesting that knockout of *OsCERK1* gene resulted in higher tolerance to CuO NPs stress than the wild-type. All these results showed that the knockout of *OsCERK1* alleviated the toxic effect of CuO NPs in rice, indicating that OsCERK1 negatively regulates the tolerance of rice to CuO NPs stress.

### 3.3. OsCERK1 Regulates Anti-Oxidative System in Response to CuO NPs Stress

**Figure 3:**
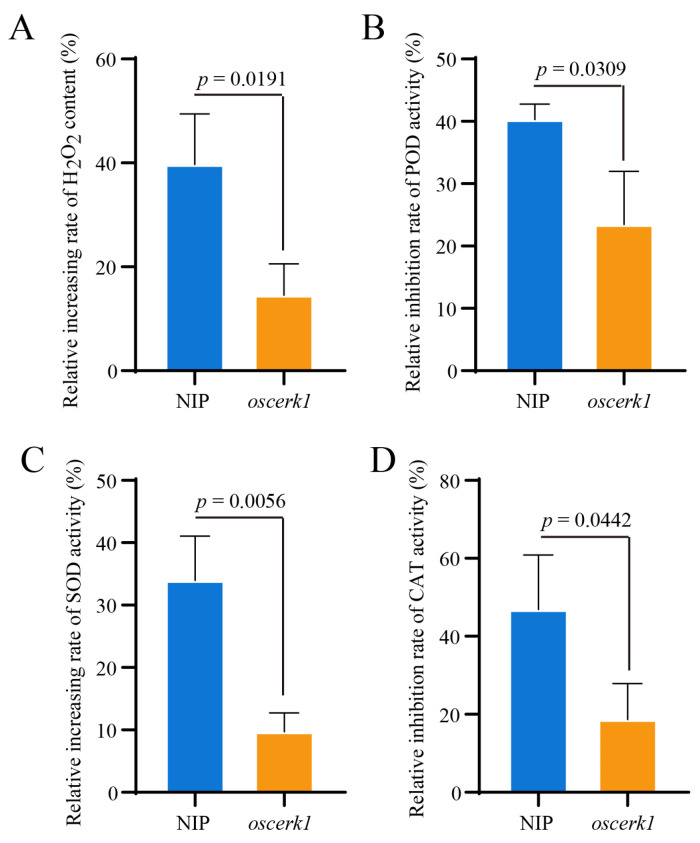
Physiological and biochemical changes of the *oscerk1* mutant upon CuO NPs treatment. (**A**) The relative increasing rates of H_2_O_2_ content in the *oscerk1* mutant and its wild-type Nipponbare (NIP) treated with CuO NPs for 14 days. The changes of POD activities (**B**), SOD activities (**C**), and CAT activities (**D**) in the *oscerk1* mutant treated with CuO NPs for 14 days. The relative increasing rate of H_2_O_2_ content was calculated based on the formula: relative increasing rate (%) = [H_2_O_2_ content (CuO NPs) − H_2_O_2_ content (control)]/H_2_O_2_ content (control) × 100%. In the same manner, the relative increasing rate of SOD activity was calculated with the formula. The relative inhibition rate of POD activity was calculated as follows: relative inhibition rate (%) = [POD activity (control) − POD activity (CuO NPs)]/POD activity (control) × 100%. Similarly, the relative inhibition rate of CAT activity was calculated with the formula. The bars are shown as means ± SD of three replications. Data was analyzed with student’s *t*-test to obtain *p* value.

In plants, the anti-oxidative system plays important roles during abiotic stress [34]. Moreover, CuO NPs treatment leads to oxidative damage in rice seedlings [35]. To further determine the functions of OsCERK1 in response to CuO NPs stress, the H_2_O_2_ levels in the *oscerk1* mutant and the wild-type seedlings treated with CuO NPs for 14 days were determined. The result showed that CuO NPs treatment highly induced the accumulation of H_2_O_2_ in the rice seedlings (Figure 3A). Compared with the wild-type, the accumulation of H_2_O_2_ in the *oscerk1* mutant was significantly reduced. The relative increasing rate of H_2_O_2_ content in the wild-type seedlings was higher than that in the *oscerk1* mutant (Figure 3A), suggesting that knockout of *OsCERK1* reduced the accumulation of H_2_O_2_ triggered by CuO NPs.

To explore why the content of H_2_O_2_ accumulation induced by CuO NPs stress in the *oscerk1* mutant was lower than that in the wild-type, the activities of anti-oxidant enzymes in the CuO NPs treated rice seedlings were determined. The results showed that CuO NPs treatment significantly down-regulated the activities of the peroxidase (POD) and catalase (CAT), which are H_2_O_2_-scavenging enzymes, in all rice seedlings (Figure 3B,D). In addition, the rate of relative inhibition rates of POD and CAT activities in the wild-type seedlings were significantly higher than those in the *oscerk1* mutant (Figure 3B,D). Furthermore, the activity of superoxide dismutase (SOD), which catalyzes the dismutation of superoxide anion (O^2−^) into oxygen and hydrogen peroxide, was significantly up-regulated by the CuO NPs treatment in the wild-type (Figure 3C). In contrast, there was no significant change in the SOD activity in the *oscerk1* mutant under CuO NPs stress condition (Figure 3C). These results indicated that knockout of *OsCERK1* relieved the negative effects of CuO NPs on the antioxidant system of rice. In conclusion, OsCERK1 negatively modulates the tolerance of rice to CuO NPs stress by down-regulating the activities of CAT and POD, and up-regulating the activity of SOD, leading to the accumulation of H_2_O_2_.

### 3.4. CuO NPs Enhance the Basal Resistance against M. oryzae in Rice

**Figure 4:**
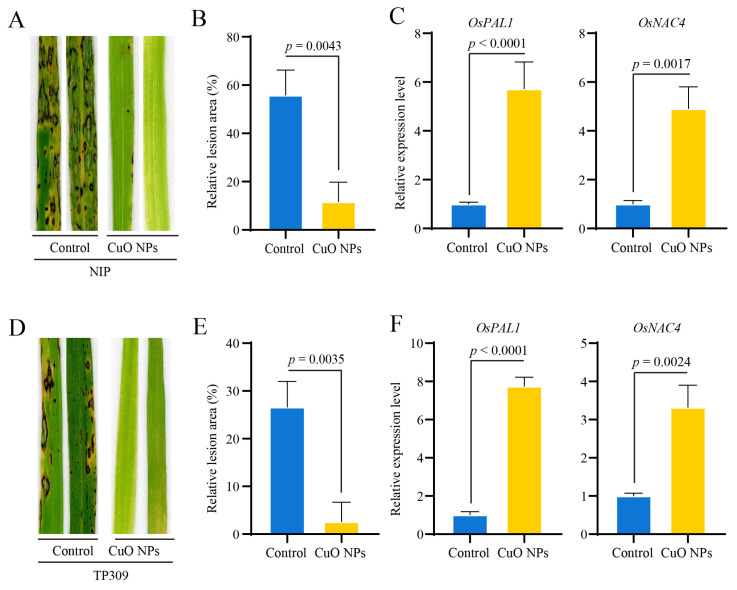
CuO NPs enhance the basal resistance against *M. oryzae* in rice. Disease symptoms (**A**), the relative lesion area (**B**), and relative expression levels of *PR* genes (**C**) of *Oryza sativa* subsp. *Japonica* cultivars Nipponbare rice seedlings inoculated with *M. oryzae* after treated with CuO NPs for 14 days. Disease symptoms (**D**), the relative lesion area (**E**), and relative expression levels of *PR* genes (**F**) of *Oryza sativa* subsp. *Japonica* cultivars TP309 rice seedlings inoculated with *M. oryzae* after treated with CuO NPs for 14 days. The relative lesion area was calculated by ImageJ. The relative transcript abundances were calculated using the 2^−ΔΔ*C*T^ method based on the abundance levels in the control samples. The *Ubiquitin* gene (*LOC_Os03g13170)* was used as the internal control for normalization. The bars are shown as means ± SD of three replications. The student’s *t*-test was used to analyze the data and generate *p* value.

Due to the role of CuO NPs in regulating the accumulation of ROS in rice, we postulated that CuO NPs stress may affect basal resistance against *M. oryzae* in rice. To confirm this hypothesis, the wild-type *Oryza sativa* subsp. *Japonica* cultivars Nipponbare and TP309 were inoculated with the *M. oryzae* wild-type strain En2-2 after treated with CuO NPs for 14 days. The seedling infection assay showed that compared with the control, the blast lesions formed on the rice seedlings with CuO NPs treatment were smaller (Figure 4A,D). Consistent with the observation of disease symptoms, the relative lesion areas calculated by ImageJ under CuO NPs were smaller than those in the control (Figure 4B,E). In addition, the relative expression levels of the *PR* genes *OsPAL1* and *OsNAC4* were up-regulated more than four folds in the Nipponbare seedlings in the response to *M. oryzae* inoculation after treated with CuO NPs for 14 days (Figure 4C). Similarly, in the TP309 seedlings, the relative expression levels of the *OsPAL1* and *OsNAC4* were also up-regulated more than three folds (Figure 4F). In conclusion, all these results demonstrated that CuO NPs treatment significantly enhances the basal resistance against *M. oryzae* in rice.

### 3.5. OsCERK1 Contributes to the CuO NPs-Modulated Basal Resistance against M. oryzae in Rice

**Figure 5:**
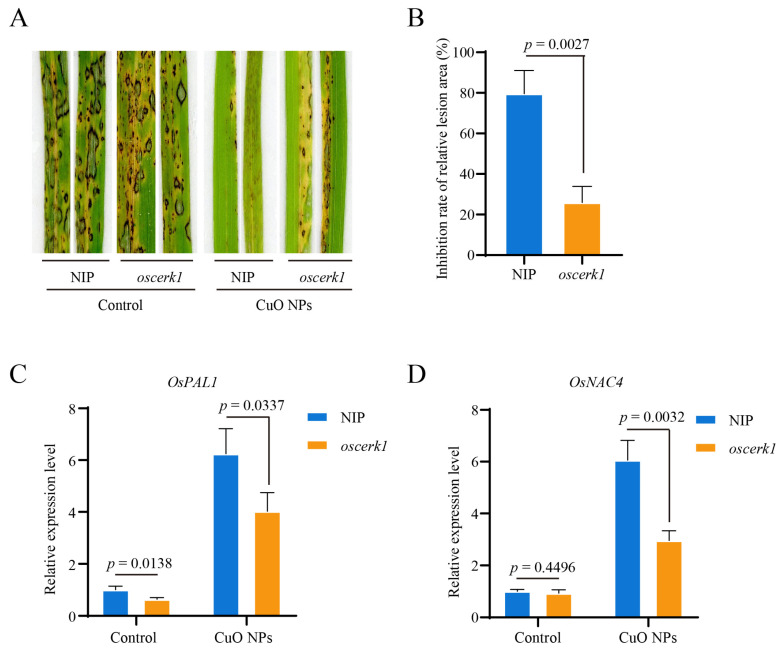
OsCERK1 contributes to basal resistance against *M. oryzae* under CuO NPs stress condition. Disease symptoms (**A**), the relative lesion areas (**B**), relative expression levels of *PR* genes *OsPAL1* (**C**) and *OsNAC4* (**D**) of the *oscerk1* mutant and its wild-type Nipponbare (NIP) inoculated with *M. oryzae* after treated with CuO NPs for 14 days. The relative lesion area was calculated by ImageJ. The relative inhibition rate of the relative lesion area was calculated based on the formula: inhibition rate (%) = [the relative lesion area (control) − the relative lesion area (CuO NPs)]/the relative lesion area (control) × 100%. The relative transcript abundances were calculated using the 2 ^−ΔΔ*C*T^ method with *Ubiquitin* gene as the internal control. The bars are shown as means ± SD of three replications. The student’s *t*-test was used to analyze the data and generate *p* value.

OsCERK1 is essential for fungal chitin-driven immune responses and contributes to basal resistance against *M. oryzae* in rice [22]. To determine whether CuO NPs-modulated basal resistance is dependent on OsCERK1 in rice, the *oscerk1* mutant and its wild-type Nipponbare were inoculated with the *M. oryzae* wild-type strain En2-2 after treated with CuO NPs for 14 days. The rice seedling infection assay showed that the blast lesions formed on the seedlings with CuO NPs treatment were significantly smaller than that without CuO NPs (Figure 5A). In contrast, compared with the wild-type, the inhibition rate of the relative lesion area caused by CuO NPs in the *oscerk1* mutant was significantly lower (Figure 5B). The rice seedling infection assay results revealed that the susceptibility of the *oscerk1* mutant to *M. oryzae* was comparable with or without CuO NPs treatment. In addition, the relative expression levels of the *PR* genes *OsPAL1* and *OsNAC4* were significantly up-regulated in all rice seedlings in response to *M. oryzae* inoculation after treated with CuO NPs for 14 days (Figure 5C,D). Compared with the wild-type, CuO NPs treatment had less effect on relative expression levels of *OsPAL1* and *OsNAC4* in the *oscerk1* mutant (Figure 5C,D). All these results suggested that OsCERK1 is required for the CuO NPs-modulated basal resistance against *M. oryzae* in rice.

## 4. Discussion

**Figure 6:**
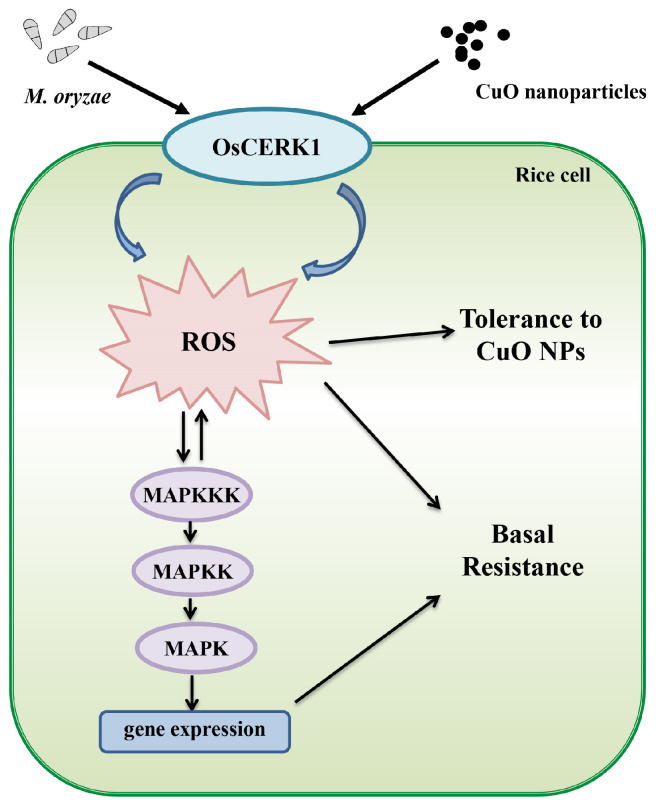
A proposed model illustrating that OsCERK1 regulates CuO NPs tolerance and CuO NPs-modulated blast resistance in rice. On the surface of rice cells, OsCERK1 senses the signals of CuO NPs stress and *M. oryzae* to regulate antioxidant system, resulting in increased ROS accumulation. The excessive ROS accumulation in the cell leads to growth inhibition but enhances the basal resistance against *M. oryzae* in rice.

With the wide application, CuO NPs pose a potential threat to cause serious toxicity to crops. In rice, CuO NPs could decrease the photosynthetic performances, increase the generation of ROS, and inhibit the growth of seedlings [36]. However, the molecular mechanisms underlying the adverse effects of CuO NPs have not yet been revealed in detail. In this study, we found that a LysM receptor-like kinase OsCERK1 negatively regulates the tolerance to CuO NPs stress. Further analyses revealed that OsCERK1 regulates the anti-oxidant system and increases the accumulation of H_2_O_2_, thus reducing the tolerance of rice to CuO NPs stress. In addition, we found that CuO NPs treatment significantly enhances the OsCERK1-mediated basal resistance against *M. oryzae* in rice. Taken together, this study demonstrated that OsCERK1 is involved in the phytotoxicity of CuO NPs and CuO NPs-modulated basal resistance against blast by regulating the activities of anti-oxidant enzymes and inducing the accumulation of ROS (Figure 6).

OsCERK1 is a critical immune receptor of rice, which is triggered by chitin and responds to fungal pathogens, such as the causal agent of rice blast disease *M. oryzae* [22]. Chitin induces oligomerization of receptors OsCEBiP and OsCERK1 to promote the phosphorylation of OsCERK1, which in turn phosphorylates and activates downstream components, thus inducing MAPK activation and ROS burst [21]. This study demonstrated that OsCERK1 is not only required for the resistance to pathogens, but also is critical for the tolerance to abiotic CuO NPs stress in rice. Similarly, AtCERK1 positively regulates disease resistance and abiotic salinity stress tolerance in *Arabidopsis* [25], which suggested that CERK1 plays an important role in the crosstalk between biotic and abiotic stress. In this study, we found that the expression of OsCERK1 is up-regulated by CuO NPs stress, leading to increase the activities of MAPK and ROS accumulation (Figure 1). Furthermore, knockout of *OsCERK1* relieved the negative impact of CuO NPs on rice growth. All these results suggested that OsCERK1 negatively regulates the tolerance to CuO NPs in rice, which is a new function of OsCERK1.

The antifungal activity of CuO NPs have been revealed [14,15,16]. For instance, CuO NPs trigger resistance in tobacco against the soil-borne fungal pathogen *Phytophthora nicotianae* [37]. However, little is known about the effect of CuO NPs on rice resistance. In this study, we found that CuO NPs significantly enhance resistance against *M. oryzae* but inhibit growth in rice. Several studies have highlighted that oxidative damage, such as lipid peroxidation, may be the main cause of CuO NPs toxicity to plants [6]. In duckweed, CuO NPs treatment increases H_2_O_2_ by 56% and •OH by 57% [17]. In *Arabidopsis*, CuO NPs (2 to 100 mg/L) induces the generation of singlet oxygen (^1^O2) and hydrogen peroxide (H_2_O_2_) in the roots and leaves [38]. In addition, the activities of CAT and SOD of the rice leaves treated with 250 mg/L CuO NPs were decreased, while the activity of the SOD in the rice roots exposed to 125 mg/L CuO NPs significantly increased [18]. Similarly, this study found that upon CuO NPs treatment, the activities of CAT and POD were significantly decreased, and the SOD activity was increased, resulting in H_2_O_2_ accumulation in rice. The induced ROS accumulation might be one of the important reasons why CuO NPs treatment significantly enhances the basal resistance against *M. oryzae* in rice. In contrast, the activities of these antioxidant enzymes and the accumulation of H_2_O_2_ in the *oscerk1* mutant with or without CuO NPs treatment were comparable. Moreover, the inhibited growth of rice seedlings caused by CuO NPs has been partially restored by the knockout of *OsCERK1*. These results indicated that OsCERK1 participates in the response to CuO NPs stress by regulating the accumulation of H_2_O_2_. It was well known that both the invasion of pathogens and CuO NPs stress could induce a burst of ROS in rice [39,40]. The reduced ROS accumulation might be one of the important reasons why the *oscerk1* mutant shows compromised resistance to *M. oryzae* and less inhibition of growth in response to CuO NPs stress. Based on these results, we inferred that in response to CuO NPs stress signal, OsCERK1 regulates the antioxidant system in rice, resulting in an increase in ROS accumulation, inhibition of growth, and enhanced resistance against *M. oryzae* in rice. In addition, these results suggested that in order to safely and properly use CuO NPs as a fungicide to prevent rice blast in the future, the phytotoxicity of CuO to rice has to be considered.

Plants face a variety of stresses in the environment. Naturally, stress signaling in plant cells is a sophisticated network. However, most studies focused on the plant response to abiotic stresses and pathogens separately in the past few centuries [41,42,43]. With the changes in climate, increasing environmental pollution, and the constant pressures of diseases and pests, the interactions between the plant response to abiotic stresses and pathogens have been attracting extensive attention worldwide. Recent studies revealed that SiO_2_ NPs can induce systemic acquired resistance in *Arabidopsis* [44], indicating that there is a crosstalk between the response to nanomaterials and disease resistance in plants. In this study, we revealed that there is a crosstalk among the signal transduction pathways of blast resistance and stress response induced by CuO NPs, with the pattern recognition receptors (PRRs) OsCERK1 as the core module. OsCERK1, which is located on the surface of rice cells, may percept not only the signals from pathogenic fungi, but also the abiotic stress signals from the environment. The results of this study revealed the molecular mechanism of OsCERK1 regulating ROS accumulation to inhibit growth and enhance blast resistance in response to CuO NPs stress in rice, as well as expanded our understanding of the crosstalk between different environmental signals.

## 5. Conclusions

This study aimed to explore the molecular mechanism of rice response to CuO NPs. The results revealed that CuO NPs treatment up-regulates the expression level of *OsCERK1*. Moreover, OsCERK1 modulates tolerance to CuO NPs in addition to basal resistance in rice. Further analyses revealed that OsCERK1 regulates the activities of anti-oxidant enzymes to induce the accumulation of ROS in the response to CuO NPs stress and contributes to CuO NPs-modulated resistance against *M. oryzae* in rice. Our results expand our understanding of the crosstalk between different environmental signals and provide important theoretical bases for the sustainable application of CuO NPs in plants.

## Data Availability

The data presented in this study are available within the article and the Supplementary Materials.

## Supplementary Materials

The following are available online at https://www.mdpi.com/article/10.3390/jof9010036/s1, Table S1: Primers used in this work.
