# Supplementary material for: OsCERK1 Contributes to Cupric Oxide Nanoparticles Induced Phytotoxicity and Basal Resistance against Blast by Regulating the Anti-Oxidant System in Rice
Source: J Fungi (Basel). 2022 Dec 26;9(1):36. doi: 10.3390/jof9010036 (PMC9866703; doi:10.3390/jof9010036)
Supplement: Supplementary file 1 [file jof-09-00036-s001.zip › jof-2071803-Supplementary.pdf]

Supplemental Table S1 Primers used in this work		
**used for**	**primer names**	**primer sequences (from 5'- to 3')**
Real-time quantitative PCR	qOsCERK1-F	GCCTCCAGAGTATGCTCGAT
	qOsCERK1-R	GAGGGCCTCCTCAAACAGAT
	qOsUBQ-F	AAGAAGCTGAAGCATCCAGC
	qOsUBQ-R	CCAGGACAAGATGATCTGCC
	qOsPAL1-F	CTACCCGCTGATGAAGAAGC
	qOsPAL1-R	GAACCTTGTTCAGCTCCTCG
	qOsNAC4-F	TCCTGCCACCATTCTGAGATG
	qOsNAC4-R	TTGCAGAATCATGCTTGCCAG
